# Retinal microvascular network attenuation in Alzheimer's disease

**DOI:** 10.1016/j.dadm.2015.04.001

**Published:** 2015-05-15

**Authors:** Michael A. Williams, Amy J. McGowan, Chris R. Cardwell, Carol Y. Cheung, David Craig, Peter Passmore, Giuliana Silvestri, Alexander P. Maxwell, Gareth J. McKay

**Affiliations:** a Centre for Medical Education, Queen's University Belfast, Belfast, UK; b Centre for Public Health, Queen's University Belfast, Belfast, UK; c Singapore Eye Research Institute, Singapore National Eye Centre, Singapore, Singapore; d Ophthalmology and Visual Sciences Academic Clinical Programme, Duke-NUS Graduate Medical School, National University of Singapore, Singapore, Singapore; e Southern Health and Social Care Trust, Craigavon Hospital, Craigavon, UK; f Centre for Experimental Medicine, Queen's University Belfast, Belfast, UK

**Keywords:** Retina, Retinal vasculature, Alzheimer's disease, Microcirculation, Small-vessel disease

## Abstract

**Introduction:**

Cerebral small-vessel disease has been implicated in the development of Alzheimer's disease (AD). The retinal microvasculature enables the noninvasive visualization and evaluation of the systemic microcirculation. We evaluated retinal microvascular parameters in a case-control study of AD patients and cognitively normal controls.

**Methods:**

Retinal images were computationally analyzed and quantitative retinal parameters (caliber, fractal dimension, tortuosity, and bifurcation) measured. Regression models were used to compute odds ratios (OR) and confidence intervals (CI) for AD with adjustment for confounders.

**Results:**

Retinal images were available in 213 AD participants and 294 cognitively normal controls. Persons with lower venular fractal dimension (OR per standard deviation [SD] increase, 0.77 [CI: 0.62–0.97]) and lower arteriolar tortuosity (OR per SD increase, 0.78 [CI: 0.63–0.97]) were more likely to have AD after appropriate adjustment.

**Discussion:**

Patients with AD have a sparser retinal microvascular network and retinal microvascular variation may represent similar pathophysiological events within the cerebral microvasculature of patients with AD.

## 1 Introduction

Alzheimer's disease (AD) is the most common form of dementia and a major increasing public health concern [1]. The gold-standard for the diagnosis of AD is the identification on post-mortem of amyloid-beta and neurofibrillary tangles [2]. It is not clear how these pathologic features result in the clinical manifestations of AD. Although AD is described as a neurodegenerative condition, it is argued that the failure of the “neurovascular unit” underlies the condition [3]. Both structural and functional cerebral vascular changes have been described in vivo and in animal models of AD [4].

The cerebral and retinal vasculature share similar embryologic origins, anatomic features, physiological properties, and regulatory mechanisms [5], and it is perhaps unsurprising that retinal changes have also been observed in AD [6], [7], [8]. Amyloid-beta has been identified in the retinal and choroidal microvasculature from mouse models of AD [9]. Berisha and colleagues reported a significant difference in the retinal venous blood column diameter between nine patients with mild to moderate AD and eight controls (*P* = .01), with AD cases having narrower veins, and a reduction in blood flow (*P* = .002) [10]. Further support arose from a much larger study, in which narrower retinal venules and a sparser and more tortuous vessel network was observed in 136 patients with AD compared with 290 matched cognitively normal controls [6].

There is now greater reliability in the assessment of a wider range of retinal parameters beyond vessel caliber, including fractal dimension, tortuosity, and vessel bifurcation which provide a global indication of the “optimality” and “efficiency” of blood distribution throughout the retinal network [11], [12], [13], [14]. These retinal features have been postulated to reflect the integrity of the cerebral microcirculation and have been associated with stroke, implicating early microvascular network abnormalities in the pathophysiology of these conditions [14], [15], [16]. Previous studies have shown association between retinal vascular changes and AD, although the effects observed have not always been either consistent or adjusted for the potential confounding of medication use. Retinal vascular changes identified in association with AD may offer value both for understanding the disease etiology and perhaps aid the early, noninvasive diagnosis of this disease [17].

The aim of this study was to compare a spectrum of retinal vascular parameters in a large sample consisting of two cohorts, one of patients with AD and another of cognitively normal controls. We hypothesize that changes within the retinal microvascular network may reflect alterations within the cerebral microcirculation of those with AD.

## 2 Methods

### 2.1 Study population

This was a prevalent case-control study comparing cases with AD to cognitively normal controls. All recruitment and testing was performed by one investigator (MW) and has been described elsewhere [18]. An opportunistic recruitment strategy was used. Potential cases with AD were identified in a nonsystematic fashion as they appeared in clinic or files from the population of those with a diagnosis of AD, made by a senior clinician using the National Institute of Neurological and Communicative Disorders and Stroke and the Alzheimer's Disease and Related Disorders Association criteria [19], attending a hospital memory clinic. Those with any other type of dementia were not included, including vascular or mixed dementia. Controls were recruited in several ways. First, carers of patients attending any out-patient clinic in the study hospital were approached as opportunities arose. Second, a university press release generated interest. Third, controls asked friends to participate. Finally, a series of talks given to AD patient support groups in the region led to volunteers. Exclusion criteria for controls were age under 65 years, Mini-Mental State Examination (MMSE) score less than 26 of 30 and unmasking of any ophthalmic history before recruitment. Testing consisted of a questionnaire, blood pressure measurement, and drawing of a blood sample to identify any confounding factors. Ethical and clinical governance approval was granted before the commencement of the study. The study followed the tenets of the Declaration of Helsinki.

### 2.2 Retinal photography and quantitative measurements of retinal microvasculature

**Fig. 1:**
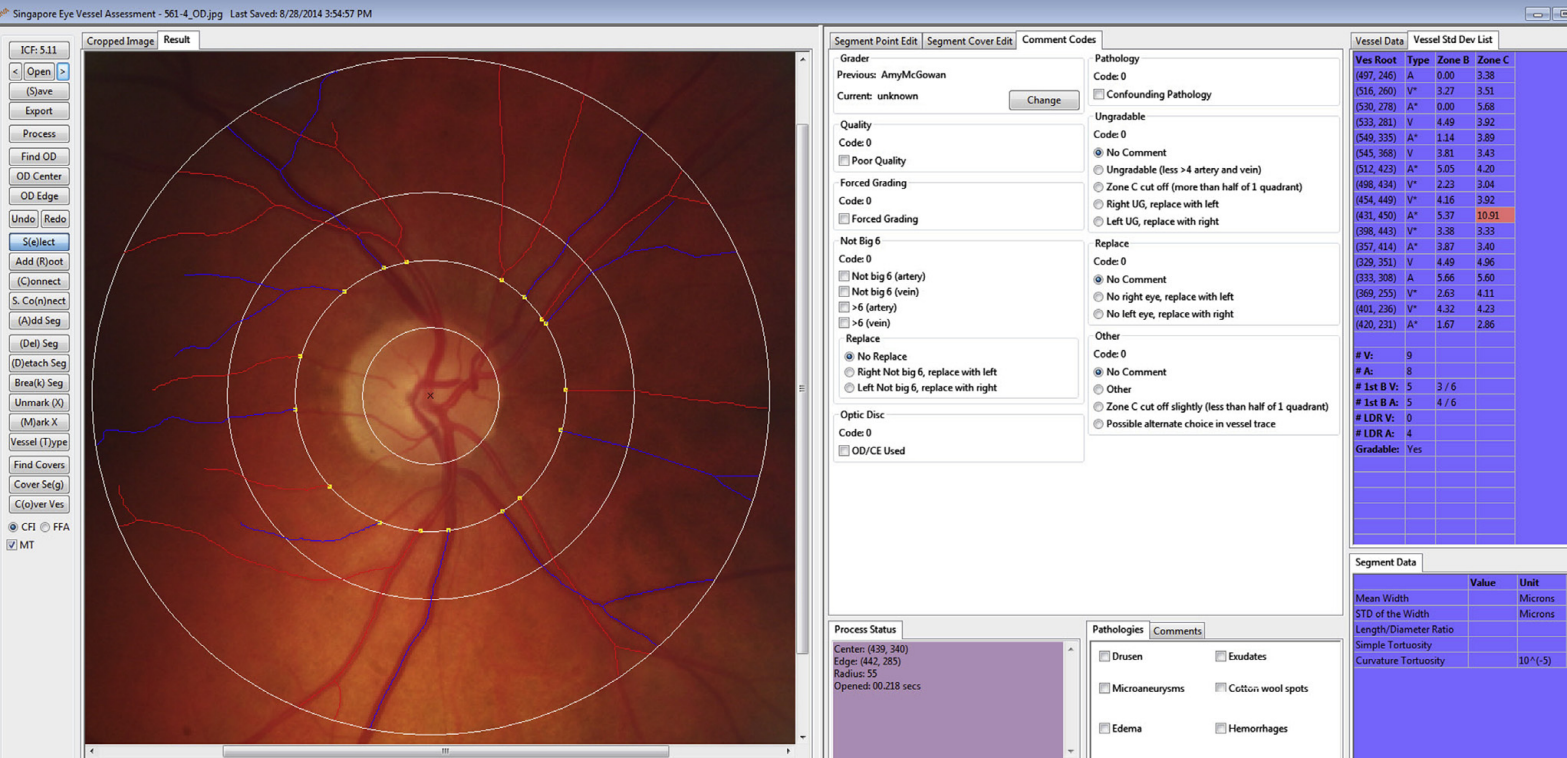
Retinal fundus image assessed quantitatively using the Singapore I Vessel Assessment (SIVA) software. Arterioles are in red and venules in blue. The measured area of retinal vascular parameters (caliber, fractal dimension, tortuosity, and branching angle) was standardized as the region from 0.5 to 2.0 optic disc diameters from the disc margin.

Retinal photography was performed through the dilated pupil using a 500 Canon CR-DGi digital camera, after the instillation of one drop of 1% tropicamide in all participants. A semiautomated computer-assisted program (Singapore I Vessel Assessment [SIVA], software version 3.0) was used to quantitatively measure the retinal vascular parameters from the photographs. SIVA automatically identifies the optic disc, places a grid with reference to the center of the optic disc, identifies vessel type, and calculates retinal vascular parameters. A single trained grader (AMG), blinded to participant characteristics, performed SIVA automated measurement and manual intervention if necessary, according to a standardized protocol [20]. The measured area was standardized and defined within the region between 0.5 and 2.0 disc diameters away from the disc margin, and all visible vessels coursing through the specified zone were measured (Fig. 1).

### 2.3 Retinal vascular caliber

Retinal vascular caliber was measured using the SIVA program following the standardized protocol used in the Atherosclerosis Risk in Communities study [21]. The retinal arteriolar and venular calibers were summarized as central retinal arterial equivalent (CRAE) and central retinal venular equivalent (CRVE), respectively, according to the revised Knudtson-Parr-Hubbard formula [22]. The reproducibility of retinal vascular measurements was high, with intragrader reliability assessed in 200 randomly selected retinal photographs and an intraclass correlation coefficient (95% confidence interval [CI]) calculated as 0.98 (CI: 0.97–0.98) for CRAE and 0.99 (CI: 0.99–0.99) for CRVE, respectively. A high correlation between the right and left eyes in retinal vascular measurements has been reported elsewhere [23]. Data from the right eye were used and when unavailable was replaced by left eye data.

### 2.4 Retinal vascular fractal dimension

Total, arteriolar, and venular fractal dimensions were determined from a skeletonized line tracing using the box counting method. These values represent a “global” summary measure of the whole branching pattern of the retinal vascular tree with larger values indicative of a more complex branching pattern [24].

### 2.5 Retinal vascular tortuosity

Retinal vascular tortuosity was estimated as the integral of the curvature square along the path of the vessel normalized by the total path length; this measure is dimensionless because it represents a ratio measure [25]. These estimates were summarized separately as retinal arteriolar and venular tortuosity. Retinal vascular tortuosity reflects the straightness/waviness of the vessels; a smaller tortuosity value is indicative of a retinal vessel with a straighter path.

### 2.6 Retinal vascular branching angle

Retinal vascular branching angle was defined as the first angle subtended between two daughter vessels at each vascular bifurcation [26]. These estimates were summarized as retinal arteriolar branching angle and retinal venular branching angle, representing the average branching angle of arterioles and venules, respectively.

### 2.7 Other variables

The following potentially confounding variables were measured, for inclusion in the conditional logistic regression modeling: age; gender; smoking (categorized as present, past, or never as per a previous study [27]); diagnosis of diabetes mellitus; diagnosis of cardiovascular disease; diagnosis of cerebrovascular disease; diagnosis of hypercholesterolemia; mean arterial blood pressure (MABP); and medication use (aspirin/clopidogrel; beta-blocker; calcium channel blocker; diuretic; nonsteroidal anti-inflammatory drug; thyroxine).

### 2.8 Statistical analysis

All statistical analyses were performed using IBM SPSS statistics version 21 (IBM Corp., Armonk, NY). An independent t test or χ^2^ test was used to compare the characteristics of AD cases and controls in the study. Quantitative retinal vascular parameters were analyzed as continuous variables (and were standardized before entry into regression models to give estimates per standard deviation [SD] increase). Logistic regression models were used to analyze the association of retinal vascular parameters with AD. Multiple logistic regression models were adjusted initially for gender, hypertension, smoking, hypercholesterolemia, diabetes mellitus, history of myocardial infarction to allow direct comparison to a previous study [6] and were then additionally adjusted for medications with a cohort frequency greater than 5% (aspirin/clopidogrel; beta-blocker; calcium channel blocker; diuretic; nonsteroidal anti-inflammatory drug; thyroxine). The models testing CRAE or CRVE related to AD were additionally adjusted for fellow vessel caliber to provide unbiased and biologically plausible results as suggested previously [28].

## 3 Results

Table 1 shows the summary characteristics of the AD (n = 213) and cognitively normal control (n = 294) groups. There were no significant differences in gender, smoking status, hypercholesterolemia, cardiovascular disease, cerebrovascular disease, or diabetes mellitus between groups. AD patients were more likely to be older than controls (79.6 vs. 76.3 yrs), and have a lower MMSE score (19.0 vs. 28.9) and MABP (95.4 vs. 101.8 mmHg) than control subjects. A significantly greater number of AD patients were more likely to be taking the following medications: aspirin/clopidogrel (48% vs. 38%), calcium channel blockers (16% vs. 10%), and diuretics (35% vs. 25%).

Gradable retinal images of sufficient quality for vessel assessment were available for all 507 participants. Table 2 shows the comparisons of retinal parameters between the AD and control groups. AD patients had significantly lower fractal dimensions (*P*_Total_ = .001; *P*_Arteriolar_ = .024; *P*_Venular_ < .001), wider (*P* = .029) but less tortuous retinal arterioles (*P* = .03). No significant variations in venular caliber, arteriolar, or venular branching angles or venular tortuosity were detected in the unadjusted analysis between both groups (*P* > .05).

Table 3 shows the associations between AD and retinal vascular parameters. In the multivariate logistic regression, persons with lower venular fractal dimension (OR per SD increase, 0.77 [CI: 0.62–0.97]) and lower arteriolar tortuosity (OR per SD increase, 0.78 [CI: 0.63–0.97]) were more likely to have AD after the adjustment for age, gender, smoking status, hypercholesterolemia, diabetes mellitus, a history of cardiovascular disease, a history of cerebrovascular disease, MABP, and medications with a frequency >5% use (aspirin/clopidogrel; beta-blocker; calcium channel blocker; diuretic; nonsteroidal anti-inflammatory drug; thyroxine). A secondary analysis conducted in all participants and separately in AD cases only failed to detect any significant associations between retinal microvascular parameters and MMSE score.

## 4 Discussion

This was the largest case-control study of which we are aware to compare retinal vascular parameters in subjects with AD and cognitively normal controls. Our study has shown that patients with AD are more likely to undergo structural changes in the retinal microvasculature manifesting in a sparser microvascular network which may reflect similar changes ongoing within the cerebral microcirculation. As expected the MMSE scores were significantly lower (*P* < .001) in the AD cohort compared with the controls and the range of scores in the AD cohort, of 1 to 29, reflected the large range of AD severity included within the study.

Overlapping risk factors for both AD and vascular disease support a role for systemic microvascular dysfunction in the pathologic changes that occur during AD pathogenesis. Retinal vessel tortuosity is reported to be the first vascular change identified in “many retinopathies” and may reflect changes in blood viscosity [29]. One study found whole blood viscosity to be significantly greater in AD cases than in age-matched controls (*P* < .05), and viscosity was related in the same study to a composite conjunctival microvascular index, based on vessel diameter, blood flow, and “microvascular abnormalities” [30]. Tortuosity is a common feature in arteries and veins frequently associated with vascular disease and aging, yet the underlying mechanisms for its initiation and development remain unclear. Multiple factors have been implicated in the process of vascular tortuosity including genetic factors, degenerative vascular disease, and alteration in blood flow and pressure, which may result in vessel buckling through the alteration of the properties of the vascular wall [31]. Mechanical instability and remodeling may offer mechanistic insight into the initiation and development of increasing blood vessel tortuosity [31].

Published data supporting a role for etiological abnormalities of the cerebral microcirculation in AD is sparse due to the difficulties associated with visualizing it. Our data suggest it is plausible changes in the retinal microvasculature in AD may represent similar pathologic changes ongoing in the cerebral microvascular network. Previously published data have identified narrower venular caliber in association with AD [6], although this effect has not always been consistently observed [32]. More recently, fractal analysis has been used to evaluate microvascular health with smaller fractal dimension values representing a sparser retinal network, more commonly associated with ill health. Several independent investigations have consistently identified a sparser retinal fractal network in association with AD [6] and cognitive impairment [33], [34] and indeed with other conditions which have an underlying microvascular component [14], [15], [16], [17].

The major strengths of our study were the large number of subjects, the range of severity of AD cases included, the number of potentially confounding factors measured, and the automated nature of measurements captured, which reduces the potential for measurement bias. Our study focused solely on AD to the exclusion of other dementia subtypes, such as vascular dementia. In addition, we have evaluated retinal fundus images using a validated computer-assisted program and standardized assessment of a range of vascular risk factors. There are several potential weaknesses to our study. First, there may be residual confounding factors not measured in our cohort that influence retinal microvascular variation but which have not been controlled for in our data. For example, changes in retinal vessel caliber can vary by up to 17% for arterioles and up to 11% for venules during one cardiac cycle [35]. Assuming that retinal images were captured randomly during the cardiac cycle, our findings related to vascular caliber, could potentially be confounded. Retinal arterial narrowing has also been reported in association with cerebral small vessel disease [36]. The degree of cerebral vascular changes in our sample of AD cases was unknown, although all had computerized tomography scans for diagnostic purposes and cardiovascular risk factors were adjusted for. Second, uncertainty in the pathology underlying clinical diagnoses of AD is a potential problem common to all ante-mortem studies on AD, despite the use of standardized clinical diagnostic criteria [37]. Third, the causal and temporal relationships between the retinal microvasculature and AD cannot be determined due to the cross-sectional nature of our study. Finally, recall bias may have led to underestimates of the prevalence of confounding factors in cases, despite a carer always being present and medical notes being consulted when needed.

Our investigation may offer further insight into the vascular contribution to the pathophysiology of AD. Pathologically ischemic changes and neurodegeneration are seen in early AD, but as both are common with increasing age, they would be expected to coexist. The relationship between cerebral vascular changes and the degenerative changes characteristic of AD is unclear: amyloid plaques may contribute to vascular damage, or vice versa [38]. In the Rotterdam study [39], cerebral hypoperfusion was associated with a greater risk of dementia although reduced cerebral blood flow may simply reflect reduced demands of an atrophying brain. Previous investigations in a mouse model of AD have suggested amyloid accumulation in vessel walls increases their rigidity and thus impairs autoregulation; the consequent reduced vascular pulsation leads to less clearance of soluble amyloid-beta [40]. Vascular dysfunction may switch on hypoxia-induced pathways, which may contribute to the development of AD through increasing amyloid-beta load [41] or tau pathology [42]. It is pertinent to note that many risk factors for AD are also vascular risk factors, such as hypertension, diabetes mellitus, and dyslipidemia. The clinical relevance of the potentially underlying role of vascular dysfunction in AD is not established. A systematic review on transcranial Doppler suggested that the method offered a noninvasive, inexpensive, and portable means to measure cerebral blood flow, and could discriminate between dementia and normal ageing, perhaps also distinguishing AD from vascular dementia [43].

Hypertension is a modifiable risk factor for dementia. Published evidence identifies impaired cerebral blood flow and whole brain atrophy as common early symptoms contributing to memory loss [44]. Despite this well-documented association, few current treatment options for dementia are directed at this potential therapeutic target [45]. Cross-sectional studies have consistently supported moderately strong associations between dementia and brain imaging abnormalities that highlight the importance of vascular disease as the underlying pathophysiology of cognitive decline [46]. Early detection is critical for the timely diagnosis of dementia and clinical intervention. However, as the earliest indicators of brain microvascular pathology are seldom detectable by magnetic resonance imaging, research to improve our understanding of AD pathogenesis, and earlier disease predictors, can improve patient care [47], [48]. Clearly the retina is easier to examine both clinically and by noninvasive imaging modalities than the brain, and retinal vascular parameters, including those measured in this study, may serve as surrogate markers in evaluating the effectiveness of novel treatments for AD.

In conclusion, the identification of retinal changes in patients with AD may aid our understanding of this condition. Future studies with preclinical AD patients may offer potential promise for the earlier identification of AD for those at increased disease risk before significant cognitive difficulties emerge.

**Research in context**

1. Systematic review: We searched PubMed until January 31, 2015, for articles published in English with the search terms “retinal microvascular abnormalities”, “dementia”, and “Alzheimer's disease”. We also reviewed reference lists of publications identified from this search, in addition to other relevant papers on retinal parameters.
2. Interpretation: Vascular mechanisms have been proposed as contributory factors in the development of Alzheimer's disease (AD). Noninvasive visualization of the human microcirculation through the retinal vasculature may reflect ongoing cerebral microvascular pathology. In this study, we have identified a sparser retinal microvascular network in patients with AD represented by a reduced venular fractal dimension and arteriolar tortuosity compared with cognitively normal controls, possibly reflecting similar alterations in cerebral microcirculation.
3. Future directions: Retinal microvascular imaging may enable better differentiation of pathophysiological dementia subtypes, improved stratification of those at increased risk of AD, and further insights into the mechanisms that contribute to the ongoing processes of AD pathology. Retinal microvascular measures may offer inexpensive surrogate measurement for evaluating future novel AD treatments for the improvement of cerebral blood flow.

## Figures and Tables

**Table 1:** Summary statistics of participants

Characteristic	Cases, n = 213	Controls, n = 294	*P*
Mean age, yrs (SD)	79.6 (7.8)	76.3 (6.6)	<.001
Mean MMSE	19.0 (5.6)	28.9 (1.2)	<.001
Male, n (%)	77 (36)	116 (40)	.45
Mean MABP (mmHg)	95.4 (10.6)	101.8 (10.6)	<.001
Ever smoked, n (%)	94 (46)	112 (38)	.09
Current smoker, n (%)	14 (7)	15 (5)	.42
Hypercholesterolemia, n (%)	80 (40)	116 (40)	.88
Cardiovascular disease, n (%)	43 (21)	70 (24)	.43
Cerebrovascular disease, n (%)	24 (12)	38 (12)	.66
Hypertension, n (%)	78 (38)	124 (42)	.34
Diabetes mellitus, n (%)	18 (9)	33 (11)	.36
Aspirin/clopidogrel, n (%)∗	98 (48)	108 (38)	.02
Beta blockers, n (%)∗	37 (18)	68 (25)	.11
Calcium channel blockers, n (%)∗	33 (16)	26 (10)	.02
Diuretics, n (%)∗	70 (35)	70 (25)	.02
NSAIDs, n (%)∗	10 (5)	20 (7)	.34
Thyroxine, n (%)∗	24 (12)	28 (10)	.47

Abbreviations: SD, standard deviation; MMSE, Mini-Mental State Examination; MABP, mean arterial blood pressure; NSAIDs, nonsteroidal anti-inflammatory drugs.

∗ Medications taken with a frequency >5%.

**Table 2:** Comparisons of retinal vascular parameters between AD cases and controls

Parameter	AD cases, n = 213	Controls, n = 294	*P*
Mean (SD)	Mean (SD)
Caliber			
Central retinal arteriolar equivalent, μm	112.8 (10.3)	110.8 (10.5)	.029
Central retinal venular equivalent, μm	159.0 (16.3)	158.9 (15.3)	.951
Fractals			
Total fractal dimension	1.396 (0.053)	1.412 (0.053)	.001
Arteriolar fractal dimension	1.194 (0.059)	1.206 (0.062)	.024
Venular fractal dimension	1.165 (0.050)	1.184 (0.050)	<.001
Tortuosity			
Arteriolar tortuosity (×10^4^)	0.876 (0.228)	0.921 (0.228)	.030
Venular tortuosity (×10^4^)	1.016 (0.276)	1.035 (0.294)	.458
Bifurcations			
Arteriolar branching angle, °	78.30 (14.82)	79.17 (15.31)	.523
Venular branching angle, °	80.38 (12.60)	79.86 (9.90)	.599

Abbreviations: AD, Alzheimer's disease; SD, standard deviation.

NOTE. *P* values were calculated by independent sample *t* test.

**Table 3:** Associations between AD and retinal vascular parameters

Parameter	Model 1	Model 2	Model 3
OR (95% CI); *P*	OR (95% CI); *P*	OR (95% CI); *P*
Caliber			
Central retinal arteriolar equivalent per SD increase∗	1.37 (1.08–1.75); .010	1.17 (0.89–1.53); .267	1.11 (0.83–1.47); .481
Central retinal venular equivalent per SD increase†	0.87 (0.69–1.11); .269	0.98 (0.74–1.29); .867	0.99 (0.75–1.32); .960
Fractals			
Total fractal dimension per SD increase	0.84 (0.70–1.02); .073	0.86 (0.70–1.06); .154	0.85 (0.68–1.06); .141
Arteriolar fractal dimension per SD increase	0.92 (0.76–1.11); .373	0.92 (0.75–1.13); .413	0.92 (0.74–1.14); .436
Venular fractal dimension per SD increase	0.75 (0.62–0.91); .004	0.78 (0.63–0.97); .024	0.77 (0.62–0.97); .025
Tortuosity			
Arteriolar tortuosity per SD increase	0.82 (0.68–0.99); .042	0.80 (0.65–0.99); .041	0.78 (0.63–0.97); .027
Venular tortuosity per SD increase	0.96 (0.80–1.16); .964	1.01 (0.83–1.24); .911	1.01 (0.82–1.24); .952
Bifurcation			
Arteriolar branching angle per SD increase	0.95 (0.79–1.14); .581	0.96 (0.78–1.18); .684	0.91 (0.73–1.14); .414
Venular branching angle per SD increase	1.08 (0.90–1.29); .404	1.03 (0.84–1.26); .791	1.10 (0.89–1.36); .389

Abbreviations: AD, Alzheimer's disease; SD, standard deviation; OR, odds ratio; CI, confidence intervals.

NOTE. Model 1 was adjusted for age and gender. Model 2 was adjusted for age, gender, mean arterial blood pressure, smoking status, hypercholesterolemia, diabetes mellitus, and history of cardiovascular disease. Model 3 was adjusted for model 2 covariates, cerebrovascular disease, and medications with a frequency >5% (aspirin/clopidogrel, beta blockers, calcium channel blockers, diuretics, nonsteroidal anti-inflammatory drugs, thyroxine).

∗ Additionally adjusted for central retinal venular equivalent.

† Additionally adjusted for central retinal arteriolar equivalent.

## References

[1] Alves L., Correia A.S., Miguel R., Alegria P., Bugalho P. (2005). Alzheimer's disease: a clinical practice-oriented review. Front Neurol.

[2] Parnell M., Guo L., Abdi M., Cordeiro M.F. (2012). Ocular manifestations of Alzheimer's disease in animal models. Int J Alzheimers Dis.

[3] Weller R.O., Boche D., Nicoll J.A. (2009). Microvasculature changes and cerebral amyloid angiopathy in Alzheimer's disease and their potential impact on therapy. Acta Neuropathol.

[4] Nicolakakis N., Hamel E. (2011). Neurovascular function in Alzheimer's disease patients and experimental models. J Cereb Blood Flow Metab.

[5] Patton N., Aslam T., MacGillivray T., Pattie A., Deary I.J., Dhillon B. (2005). Retinal vascular image analysis as a potential screening tool for cerebrovascular disease: a rationale based on homology between cerebral and retinal microvasculatures. J Anat.

[6] Cheung C.Y., Ong Y.T., Ikram M.K., Ong S.Y., Li X., Hilal S. (2014). Microvascular network alterations in the retina of patients with Alzheimer's disease. Alzheimers Dement.

[7] Cheung C.Y., Ong Y.T., Ikram M.K., Chen C., Wong T.Y. (2014). Retinal microvasculature in Alzheimer's disease. J Alzheimers Dis.

[8] Frost S., Kanagasingam Y., Sohrabi H., Vignarajan J., Bourgeat P., Salvado O. (2013). Retinal vascular biomarkers for early detection and monitoring of Alzheimer's disease. Transl Psychiatry.

[9] Ning A., Cui J., To E., Ashe K.H., Matsubara J. (2008). Amyloid-beta deposits lead to retinal degeneration in a mouse model of Alzheimer disease. Invest Ophthalmol Vis Sci.

[10] Berisha F., Feke G.T., Trempe C.L., McMeel J.W., Schepens C.L. (2007). Retinal abnormalities in early Alzheimer's disease. Invest Ophthalmol Vis Sci.

[11] Patton N., Pattie A., MacGillivray T., Aslam T., Dhillon B., Gow A. (2007). The association between retinal vascular network geometry and cognitive ability in an elderly population. Invest Ophthalmol Vis Sci.

[12] Murray C.D. (1926). The physiological principle of minimum work: I. The vascular system and the cost of blood volume. Proc Natl Acad Sci U S A.

[13] Sherman T.F. (1981). On connecting large vessels to small. The meaning of Murray's law. J Gen Physiol.

[14] Kawasaki R., Che Azemin M.Z., Kumar D.K., Tan A.G., Liew G., Wong T.Y. (2011). Fractal dimension of the retinal vasculature and risk of stroke: a nested case-control study. Neurology.

[15] Cheung N., Liew G., Lindley R.I., Liu E.Y., Wang J.J., Hand P. (2010). Retinal fractals and acute lacunar stroke. Ann Neurol.

[16] Doubal F.N., MacGillivray T.J., Patton N., Dhillon B., Dennis M.S., Wardlaw J.M. (2010). Fractal analysis of retinal vessels suggests that a distinct vasculopathy causes lacunar stroke. Neurology.

[17] Heringa S.M., Bouvy W.H., van den Berg E., Moll A.C., Kappelle L.J., Biessels G.J. (2013). Associations between retinal microvascular changes and dementia, cognitive functioning, and brain imaging abnormalities: a systematic review. J Cereb Blood Flow Metab.

[18] Williams M.A., Silvestri V., Craig D., Passmore A.P., Silvestri G. (2014). The prevalence of age-related macular degeneration in Alzheimer's disease. J Alzheimers Dis.

[19] McKhann G., Drachman D., Folstein M., Katzman R., Price D., Stadlan E.M. (1984). Clinical diagnosis of Alzheimer's disease: report of the NINCDS-ADRDA Work Group under the auspices of Department of Health and Human Services Task Force on Alzheimer's Disease. Neurology.

[20] Cheung C.Y., Tay W.T., Mitchell P., Wang J.J., Hsu W., Lee M.L. (2011). Quantitative and qualitative retinal microvascular characteristics and blood pressure. J Hypertens.

[21] Hubbard L.D., Brothers R.J., King W.N., Clegg L.X., Klein R., Cooper L.S. (1999). Methods for evaluation of retinal microvascular abnormalities associated with hypertension/sclerosis in the Atherosclerosis Risk in Communities Study. Ophthalmology.

[22] Knudtson M.D., Lee K.E., Hubbard L.D., Wong T.Y., Klein R., Klein B.E. (2003). Revised formulas for summarizing retinal vessel diameters. Curr Eye Res.

[23] Cheung C.Y., Hsu W., Lee M.L., Wang J.J., Mitchell P., Lau Q.P. (2010). A new method to measure peripheral retinal vascular caliber over an extended area. Microcirculation.

[24] Liew G., Wang J.J., Cheung N., Zhang Y.P., Hsu W., Lee M.L. (2008). The retinal vasculature as a fractal: methodology, reliability, and relationship to blood pressure. Ophthalmology.

[25] Hart W.E., Goldbaum M., Cote B., Kube P., Nelson M.R. (1999). Measurement and classification of retinal vascular tortuosity. Int J Med Inform.

[26] Zamir M., Medeiros J.A., Cunningham T.K. (1979). Arterial bifurcations in the human retina. J Gen Physiol.

[27] Tomany S.C., Wang J.J., van Leeuwen R., Klein R., Mitchell P., Vingerling J.R. (2004). Risk factors for incident age-related macular degeneration: pooled findings from 3 continents. Ophthalmology.

[28] Liew G., Sharrett A.R., Kronmal R., Klein R., Wong T.Y., Mitchell P. (2007). Measurement of retinal vascular caliber: issues and alternatives to using the arteriole to venule ratio. Invest Ophthalmol Vis Sci.

[29] Benitez-Aguirre P.Z., Sasongko M.B., Craig M.E., Jenkins A.J., Cusumano J., Cheung N. (2012). Retinal vascular geometry predicts incident renal dysfunction in young people with type 1 diabetes. Diabetes Care.

[30] Smith M.M., Chen P.C., Li C.S., Ramanujam S., Cheung A.T. (2009). Whole blood viscosity and microvascular abnormalities in Alzheimer's disease. Clin Hemorheol Microcirc.

[31] Han H.C. (2012). Twisted blood vessels: symptoms, etiology and biomechanical mechanisms. J Vasc Res.

[32] de Jong F.J., Schrijvers E.M., Ikram M.K., Koudstaal P.J., de Jong P.T., Hofman A. (2011). Retinal vascular caliber and risk of dementia: the Rotterdam study. Neurology.

[33] Ong Y.T., Hilal S., Cheung C.Y., Xu X., Chen C., Venketasubramanian N. (2014). Retinal vascular fractals and cognitive impairment. Dement Geriatr Cogn Dis Extra.

[34] Cheung C.Y., Ong S., Ikram M.K., Ong Y.T., Chen C.P., Venketasubramanian N. (2014). Retinal vascular fractal dimension is associated with cognitive dysfunction. J Stroke Cerebrovasc Dis.

[35] Knudtson M.D., Klein B.E., Klein R., Wong T.Y., Hubbard L.D., Lee K.E. (2004). Variation associated with measurement of retinal vessel diameters at different points in the pulse cycle. Br J Ophthalmol.

[36] Kwa V.I., van der Sande J.J., Stam J., Tijmes N., Vrooland J.L. (2002). Amsterdam Vascular Medicine Group. Retinal arterial changes correlate with cerebral small-vessel disease. Neurology.

[37] Knopman D.S., DeKosky S.T., Cummings J.L., Chui H., Corey-Bloom J., Relkin N. (2001). Practice parameter: diagnosis of dementia (an evidence-based review). Report of the Quality Standards Subcommittee of the American Academy of Neurology. Neurology.

[38] Iadecola C. (2010). The overlap between neurodegenerative and vascular factors in the pathogenesis of dementia. Acta Neuropathol.

[39] Ruitenberg A., den Heijer T., Bakker S.L., van Swieten J.C., Koudstaal P.J., Hofman A. (2005). Cerebral hypoperfusion and clinical onset of dementia: the Rotterdam Study. Ann Neurol.

[40] Okamoto Y., Yamamoto T., Kalaria R.N., Senzaki H., Maki T., Hase Y. (2012). Cerebral hypoperfusion accelerates cerebral amyloid angiopathy and promotes cortical microinfarcts. Acta Neuropathol.

[41] Sun X., He G., Qing H., Zhou W., Dobie F., Cai F. (2006). Hypoxia facilitates Alzheimer's disease pathogenesis by up-regulating BACE1 gene expression. Proc Natl Acad Sci U S A.

[42] Koike M.A., Garcia F.G., Kitazawa M., Green K.N., Laferla F.M. (2011). Long term changes in phospho-APP and tau aggregation in the 3xTg-AD mice following cerebral ischemia. Neurosci Lett.

[43] Keage H.A., Churches O.F., Kohler M., Pomeroy D., Luppino R., Bartolo M.L. (2012). Cerebrovascular function in aging and dementia: a systematic review of transcranial Doppler studies. Dement Geriatr Cogn Dis Extra.

[44] Kehoe P.G., Passmore P.A. (2012). The renin-angiotensin system and antihypertensive drugs in Alzheimer's disease: current standing of the angiotensin hypothesis?. J Alzheimers Dis.

[45] Goodison W.V., Frisardi V., Kehoe P.G. (2012). Calcium channel blockers and Alzheimer's disease: potential relevance in treatment strategies of metabolic syndrome. J Alzheimers Dis.

[46] van de Haar H.J., Burgmans S., Hofman P.A., Verhey F.R., Jansen J.F., Backes W.H. (2014). Blood-brain barrier impairment in dementia: current and future in vivo assessments. Neurosci Biobehav Rev.

[47] Hanff T.C., Sharrett A.R., Mosley T.H., Shibata D., Knopman D.S., Klein R. (2014). Retinal microvascular abnormalities predict progression of brain microvascular disease: an atherosclerosis risk in communities magnetic resonance imaging study. Stroke.

[48] Sivak J.M. (2013). The aging eye: common degenerative mechanisms between the Alzheimer's brain and retinal disease. Invest Ophthalmol Vis Sci.

